# Comparing Mitochondrial Activity, Oxidative Stress Tolerance, and Longevity of Thirteen *Ascomycota* Yeast Species

**DOI:** 10.3390/antiox12101810

**Published:** 2023-09-28

**Authors:** Anna Gröger, Ilune Martínez-Albo, M. Mar Albà, José Ayté, Montserrat Vega, Elena Hidalgo

**Affiliations:** 1 Oxidative Stress and Cell Cycle Group, Universitat Pompeu Fabra, C/Doctor Aiguader 88, 08003 Barcelona, Spain; anna.groeger@uk-koeln.de (A.G.); ilunemartinez2000@gmail.com (I.M.-A.); jose.ayte@upf.edu (J.A.); 2 Evolutionary Genomics Group, Research Programme on Biomedical Informatics, Hospital del Mar Research Institute (IMIM), C/Doctor Aiguader 88, 08003 Barcelona, Spain; malba@imim.es; 3 Catalan Institute for Research and Advanced Studies (ICREA), 08010 Barcelona, Spain

**Keywords:** aging, *S. pombe*, *S. cerevisiae*, *Ascomycota*, mitochondrial activity, respiration, H_2_O_2_ tolerance

## Abstract

Aging is characterized by a number of hallmarks including loss of mitochondrial homeostasis and decay in stress tolerance, among others. Unicellular eukaryotes have been widely used to study chronological aging. As a general trait, calorie restriction and activation of mitochondrial respiration has been proposed to contribute to an elongated lifespan. Most aging-related studies have been conducted with the Crabtree-positive yeasts *Saccharomyces cerevisiae* and *Schizosaccharomyces pombe*, and with deletion collections deriving from these conventional yeast models. We have performed an unbiased characterization of longevity using thirteen fungi species, including *S. cerevisiae* and *S. pombe*, covering a wide range of the *Ascomycota* clade. We have determined their mitochondrial activity by oxygen consumption, complex IV activity, and mitochondrial redox potential, and the results derived from these three methodologies are highly overlapping. We have phenotypically compared the lifespans of the thirteen species and their capacity to tolerate oxidative stress. Longevity and elevated tolerance to hydrogen peroxide are correlated in some but not all yeasts. Mitochondrial activity per se cannot anticipate the length of the lifespan. We have classified the strains in four groups, with members of group 1 (*Kluyveromyces lactis*, *Saccharomyces bayanus* and *Lodderomyces elongisporus*) displaying high mitochondrial activity, elevated resistance to oxidative stress, and elongated lifespan.

## 1. Introduction

During the last decades, there has been a growing interest in investigating the molecular bases of aging and aging-associated disorders. Health and fitness as opposed to aging have been reviewed by researchers, to try to simplify or provide unifying hallmarks of these very complex processes such as health and decay. While the maintenance of an optimal and healthy lifestyle has been postulated as the absence of pathology, some hallmarks can disrupt homeostasis and characterize the aging process.

There is still a need to completely characterize and understand the molecular basis of this progressive loss of physiological integrity which defines aging. The literature is very complex regarding the definition of this time-dependent decline; several reviews by world-wide leaders in the aging field defined and are continuously updating the list of unifying hallmarks characterizing this decay of physiological events. Thus, twelve hallmarks such as loss of proteostasis or mitochondrial dysfunction are common to aging [1], and these marks are interconnected with the main proposed indicators of health, namely spatial compartmentalization, maintenance of homeostasis, and adequate responses to stress [2].

The molecular mechanisms that govern longevity are highly conserved throughout evolution, highlighting the importance of model organisms, such as yeast, in studying this process. The unicellular eukaryote *Saccharomyces cerevisiae* has been widely used as a model system to define the molecular bases of the aging process. Studies using this budding yeast have made it possible to identify signaling pathways and media conditions, which regulate fitness and lifespan extension. Recently, the fission yeast *Schizosaccharomyces pombe* has emerged as a challenging and complementary aging model.

There are two different aging models defined in yeast. The replicative lifespan (RLS) is a model referred to mitotically active cells, in which lifespan is defined by the number of daughter cells that a mother cell can produce before senescence. The chronological lifespan (CLS) is defined as the survival of non-proliferating cells. CLS is commonly measured by culturing cells in liquid media, where they enter a non-dividing state once some nutrients have been exhausted; loss of viability at this stage is a clear hallmark of CLS. This method determines how long yeast cells in culture can survive in a non-dividing phase; most nutrients remain available during the initial stationary phase, and therefore cell death is not caused by full nutrient exhaustion. While *S. cerevisiae* has allowed the identification of genes and pathways required for longevity regarding both RLS and CLS, the symmetric division of *S. pombe* complicates RLS studies and has been used to identify growth conditions and proteins regulating CLS (for reviews on yeasts’ RLS and CLS, see [3,4]).

Regarding CLS, calorie restriction increases longevity in both yeasts and in other model organisms, being considered the most common anti-aging intervention. The effect of calorie restriction is most likely mediated by the inhibition of highly conserved nutrient-responsive kinases, TOR and PKA, with the direct or indirect activation of stress-regulated kinases such as Sty1 in *S. pombe* [5] (for reviews on stress and aging in yeast, see [6,7]).

Mitochondrial metabolism plays a key role in the regulation of CLS and is probably a downstream effector of calorie restriction. Thus, up-regulation of mitochondrial respiration has been linked to the activation of stress responses and lifespan promotion. Under non-restrictive nutrient conditions, *S. pombe* obtains energy from both fermentation and respiration, but under glucose-depleted conditions, more efficient mitochondrial respiratory metabolism is induced; a mild reduction in glucose concentration from 3% to 1% in rich fission yeast media is sufficient to promote longevity. Under this mild glucose starvation, cells display increased respiratory metabolism and enhanced turnover of defective mitochondria by mitophagy [8].

However, while respiratory metabolism has been associated with a beneficial effect on the longevity of calorie restriction, mitochondrial dysfunction occurring upon excessive use or upon loss of mitochondrial quality control or reduced mitophagy is an accepted hallmark of aging (for reviews, see [1,9,10,11]).

Yeasts play important roles in different natural environments as well as in artificial biotechnological processes, and they have diverse and great importance as model systems. Comparing several of them has become crucial to investigate in detail the relationships between genome sequences, biological capacities and specific phenotypes [12]. The information gathered from many yeast species, isolated from diverse natural environments and with an extremely long evolutionary history, can usefully complement the information about basic biological mechanisms obtained from *S. cerevisiae* and *S. pombe.* With the aim of increasing our knowledge of the molecular events controlling health span and to find new model systems to analyze the role of stress responses and mitochondrial respiration in CLS, we have grown 13 different yeasts (12 of the hemiascomycete/budding yeasts subdivision and the archiascomycete *S. pombe* [13]) in the same high glucose media and analyzed the relationship among (i) longevity, (ii) mitochondrial activity, and (iii) tolerance to oxidative stress. While the relative contribution of respiratory capacity does not seem to directly contribute to longevity, there is correlation between sustained viability during CLS and tolerance to hydrogen peroxide (H_2_O_2_) during the logarithmic phase in some species. We propose that the capacity to respond to reactive oxygen species directly contributes to enhanced lifespan.

## 2. Materials and Methods

### 2.1. Strains Used in This Study

*S. pombe* (972 (*h*^−^; [14]) and AV18 (*h^−^ sty1::kanMX6*; [15])) and *S. cerevisiae* (BY4741 (*MATa*, *his3Δ1 leu2Δ0 met15Δ0 ura3Δ0*; Invitrogen, Waltham, MA, USA) and W303 [16]) strains are from our lab stocks. Strains *Saccharomyces (Lachancea) kluyveri*, *Kluyveromyces (Lachancea) thermotolerance*, *Lachancea (Kluyveromyces) waltii*, *Kluyveromyces lactis*, *Saccharomyces (Naumovia) castellii*, *Saccharomyces bayanus*, *Saccharomyces kudriavzevii*, *Saccharomyces mikatae* and *Saccharomyces paradoxus*, were obtained from the Verstrepen and Carey labs, as already indicated [17]. *Lodderomyces elongisporus* and *Yarrowia lipolytica* were also obtained from the same sources. All yeasts were grown in *S. pombe* rich medium, namely YE5S [18], in YPD, or in custom rich YPD [19] when indicated.

### 2.2. Measuring Chronological Lifespan by Flow Citometry

We prepared pre-cultures of all 13 yeasts in YE5S and inoculated YE5S cultures overnight to reach an OD_600_ of 0.5 (logarithmic phase). We measured cell viability at the logarithmic phase and took samples every 2 days (days 2, 4, 6, and 8) of stationary growth. Cell viability was measured using propidium iodide (PI) as described previously [20]. Briefly, 2 × 10^7^ cells were collected and resuspended in 1 mL of PBS with 2 µM of PI dur-ing 30 min at 30 °C in the dark. Ten thousand cells were analyzed by flow cytometry, using FACSCanto^TM^ (BD Biosciences, Franklin Lakes, NY, USA). PI staining was monitored using the PE-A channel to detect red fluorescence. We extracted the data of the percentage of living cells to build survival curves, considering that the logarithmic phase sample contains 100% of viable cells.

### 2.3. Oxygen Consumption

Oxygen consumption was performed as described previously [21]. Briefly, cells were collected at their logarithmic phase and 2 × 10^7^ cells were resuspended in 1 mL of YE5S to a final OD_600_ of 1. The measurements were made using Oxytherm + R system (Hansatech, Pentney, UK), with the standard setting to measure in nmoles/mL, recorded over 10 min. The addition of carbonyl cyanide 4-(trifluoromethoxy)phenylhydrazone (FCCP) (6 µM) induced maximal respiration by uncoupling electron transport from oxidative phosphorylation. Each one of the measurements was performed from biological triplicates.

### 2.4. Preparation of Mitochondria-Enriched Fractions

Mitochondria were purified from protoplasts prepared using the Zymolyase and lysing enzymes from *Trichoderma harzianum* as described before [8,22]. Briefly, protoplasts were broken by 10 strokes using a glass homogenizer, and mitochondria-enriched fractions were obtained after two centrifugation steps at 800× *g* and 12,000× *g*.

### 2.5. Citrate Synthase Activity

Citrate synthase activity was used to normalize the mitochondrial-enriched fractions, and was measured using a coupled enzyme reaction, which results in a colorimetric (412 nm) product proportional to the enzymatic activity present, as described previously [23]. Briefly, 10 µg of mitochondria-enriched fractions were incubated with 80 mM acetyl-CoA, 0.5 mM 5,5-dithio-bis(2-nitrobenzoic acid) (DNTB), in 1 mL solution containing 100 mM Tris pH 8.0. Then, 0.5 mM oxaloacetic acid was added. Absorbance at 412 nm was measured every 30 s during 2 min at 25 °C. We used an extinction coefficient for 2-nitro-5-thiobenzoate (TNB) of 13.6/mM/cm. The activity was expressed in units per mg (µmol TNB/min/mg mitochondria-enriched fraction).

### 2.6. Measurement of Complex IV Activity in Mitochondria-Enriched Protein Fractions

Complex IV activity in mitochondria-enriched fractions was measured as described previously [24]. Briefly, horse heart cytochrome c was dissolved in assay buffer (50 mM phosphate buffer pH 7.4 and 120 mM KCl) to a final concentration of ~1 mM, and reduced with 0.5 mM DTT. Reduced cytochrome c was loaded on a gel filtration chromatography column (Sephadex G25) to eliminate DTT, yielding a solution of ~300 µM. Complex IV activity measurements were performed by incubation of mitochondria-enriched fractions with reduced cytochrome c (final concentration of 30 µM) in assay buffer. To ensure reaction linearity, cytochrome c oxidation activity was initiated by the addition of 2–10 µg of mitochondria-enriched fractions, depending on the complex IV activity of each specific sample. The rates of cytochrome c oxidation were measured spectrophotometrically at 550 nm. The extinction coefficient for the oxidation of cytochrome c is 19.6/mM/cm. The activity was expressed in nanomoles of cytochrome c in oxidized form/minute/unit of citrate synthase activity (see above).

### 2.7. Tolerance to H_2_O_2_ in Liquid Cultures—Recording of Growth Curves

Yeast cells were grown in YE5S at 30 °C to an OD_600_ of 0.5 in flasks, then cultures were diluted to an OD_600_ of 0.05 and growth proceeded in flasks for 20–30 min, to an OD_600_ of 0.1. We then transferred 100-µL samples to 96-well plates in duplicates, added or not 2 mM or 10 mM H_2_O_2_, and OD_600_ were automatically recorded to generate growth curves using a the Power Wave microplate scanning spectrophotometer (Bio-Tek, Winooski, VT, USA) and Gen5 software version 1.04.5, as previously described [25]. We calculated the time of “arrest after H_2_O_2_” by subtracting the minutes required to reach an absorbance at 600 nm of 0.5 between 2 mM H_2_O_2_-treated and untreated cultures, as previously described [26].

### 2.8. Microscopy and Image Analysis

Samples from cell cultures in the logarithmic phase grown in YE5S were harvested by centrifugation and were visualized at room temperature, as previously described [8]. Cell cultures at OD_600_ 0.5 were incubated with 0.1 μg/mL MitoTracker Red CMXRos (Invitrogen, Waltham, MA, USA) for 30 min, as described before [8]. Briefly, cells were washed, centrifuged, and resuspended in YE5S with 3% glucose. Images were acquired using a Nikon Eclipse 90i microscope equipped with differential interference contrast optics, a PLAN APO VC 100 × 1.4 oil immersion objective, an ORCA-II-ERG camera (Hamamatsu, Iwata, Japan), an excitation and emission filter mCherry-C (IDEX/Semrock, Rochester, NY, USA), and image acquisition software Metamorph 7.8.13 (Gataca Systems). The processing of all images was performed using Fiji (ImageJ, version 1.54f, National Institutes of Health) [27].

### 2.9. Statistics

Unless otherwise stated, all experiments were performed at least three times and representative experiments were shown. Significant differences were determined using a two-sided *t*-test.

## 3. Results

### 3.1. Growth of 13 Species of Yeast in S. pombe Rich Medium YE5S

**Figure 1:**
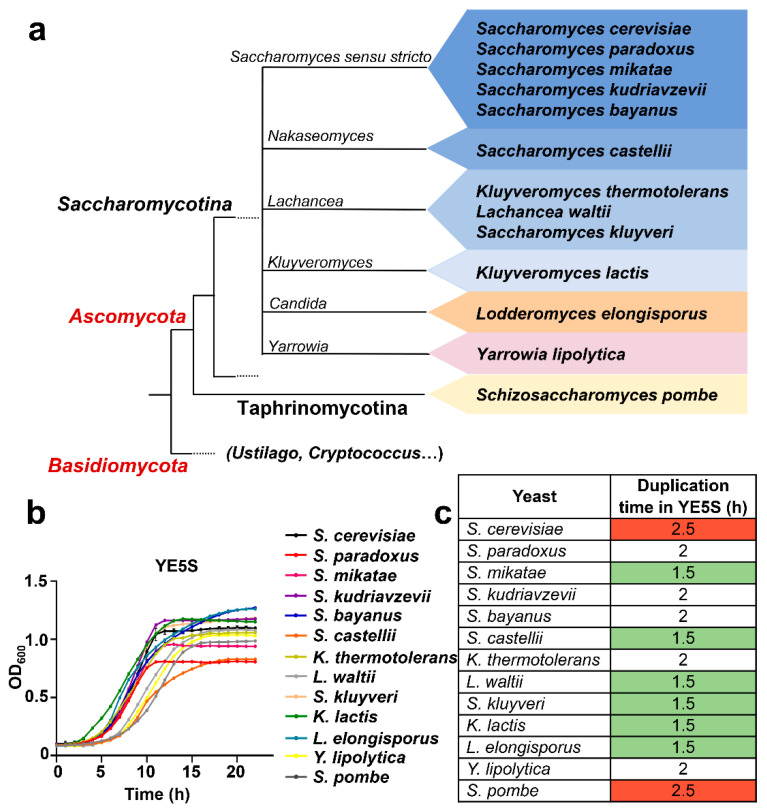
Growth curves and duplication times of 13 yeast species in YE5S. (**a**) Scheme depicting evolutionary distance of yeast species belonging to the *Saccharomycetae* family (blue), candida clade (orange), *Diapodascacea* (pink) and *Schizosaccharomycetaceae* (yellow) families. (**b**) The 13 different yeasts display comparable growth rates in YE5S media. Cell growth was monitored measuring OD_600_ during 24 h. Each line represents the average of three biological triplicates, with error bars (standard deviation, SD) displayed in Figure S1. (**c**) Table showing the duplication time in hours of the indicated yeast in YE5S media. Color indicates relative short (green) or long (red) duplication times.

With the aim of analyzing longevity, mitochondrial activity and stress tolerance of eukaryotic representatives of non-distant evolutionary phyla, we chose to compare 13 different yeasts: 12 hemiascomycetes and the distantly related *S. pombe* (in yellow in Figure 1a). Among the 12 *Sacccharomycotina* species, they are distributed into three major groups: the Saccharomycetaceae family (in blue in Figure 1a; *S. cerevisiae*, *S. paradoxus*, *S. mikatae*, *S. kudriavzevii*, *S. bayanus*, *S. castellii*, *K. thermotolerans*, *L. waltii*, *S. kluyveri*, *K. lactis*); the ‘CTG group’ (in orange in Figure 1a; *L. elongisporus*); and the Dipodascaceae family (in pink in Figure 1a; *Y. lipolytica*) [28].

We first tested whether a common rich media would sustain the growth of the different yeasts with comparable duplication times and maximal cell densities. We recorded the growth curves in 96-well plates under shaking at 30 °C of the 13 yeasts in *S. pombe* rich medium (YE5S) [29], *S. cerevisiae* YPD medium [30], and a modified, custom rich YPD [19]. As shown in Figure 1b (growth in YE5S) and Figure S1 (growth in YPD and custom rich YPD), yeasts displayed similar kinetics in all media, with YE5S allowing more homogeneous behaviors regarding maximal optical densities and duplication times. As shown in Figure 1c, *S cerevisiae* and *S. pombe* displayed the longer duplication times in YE5S (in red in Figure 1c; 2.5 h), while six yeasts species doubled almost twice as fast (in green in Figure 1c; 1.5 h).

We decided to continue with our analysis of these yeasts using YE5S as a unifying growth medium.

### 3.2. Analysis of CLS in the 13 Yeast Species—Measuring Viability at the Stationary Phase

**Figure 2:**
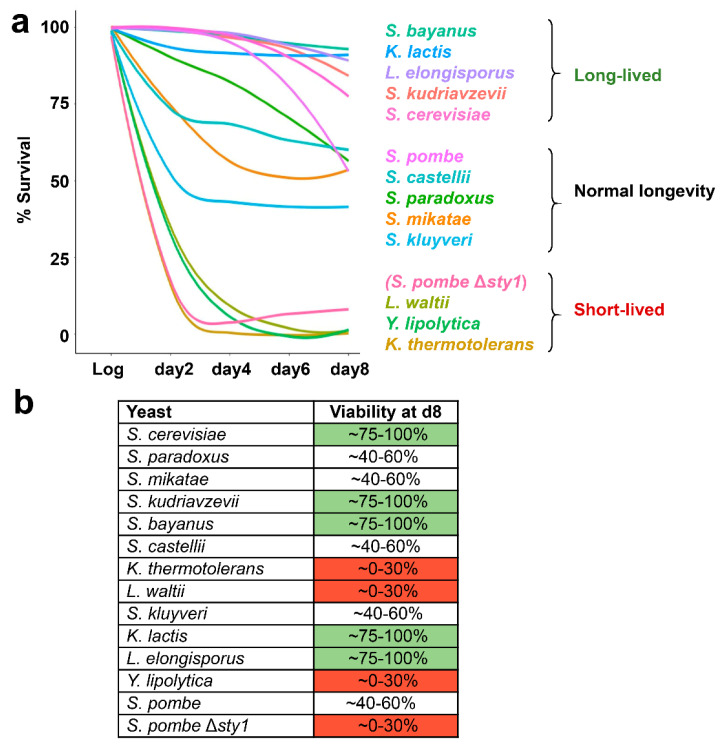
Determination of the CLS of the 13 yeast species by flow cytometry. (**a**) Longevity of each yeast strain was measured in YE5S media for 8 days. Each line represents the percentage of survival at logarithmic growth (Log), day 2, day 4, day 6 and day 8 of stationary phase. The strain Δ*sty1* of *S. pombe* was used as a short-lived control. (**b**) Classification of the yeasts by their lifespan. In the second column the percentage of viable cells at day 8 is indicated. Long-lived strains are colored in green (*S. cerevisiae*, *S. kudiavzevii*, *S. bayanus*, *K. lactis*, and *L. elongisporus*), short-lived strains marked in red (*K. thermotolerans*, *L. waltii*, *Y. lipolityca* and *S. pombe* Δ*sty1*) and regular longevity in white (*S. parodoxus*, *S. mikatae*, *S. castellii*, *S. kluyveri* and *S. pombe*).

To determine cell viability during chronological aging, we monitored the uptake and accumulation of a fluorescent dye, propidium iodide, by flow cytometry, as done before in *S. pombe* [8]. We used wild-type and the MAP kinase-lacking Δ*sty1* fission yeast strains as standards of average lifespan and short-lived cultures, respectively; cells lacking the MAP kinase Sty1 display reduced viability at the stationary phase [5]. Starting with cultures at their logarithmic phase, we then took cell culture samples every two days until day 8. Cells from logarithmic and aged cultures were stained with propidium iodide and analyzed by flow cytometry to quantify the non-stained viable population. We show in Figure 2a and Figure S2 the survival curve of each yeast species, by representing the percentage of viable cells at each time point.

In general, the initial decay of survival at day 2 and the low viability at day 8 were concomitantly affected for each yeast type. We classified the 13 species in three groups, based on their viability at day 8: long-lived (~75–100% survival; in green in Figure 2b), displaying normal longevity (using wild-type *S. pombe* as a reference; ~40–60% survival), and short-lived (using Δ*sty1 S. pombe* as a reference; ~0–30% survival; in red in Figure 2b).

### 3.3. Measuring Mitochondrial Activity in the Different Yeasts

Once we had reported differences in the lifespans of the different 13 yeasts, we determined mitochondrial respiration by three different strategies: (i) measuring oxygen consumption of cultures at the logarithmic phase, (ii) recording complex IV activity of mitochondrial-enriched fractions, and (iii) staining with MitoTracker Red CMXRos of cell suspensions.

**Figure 3:**
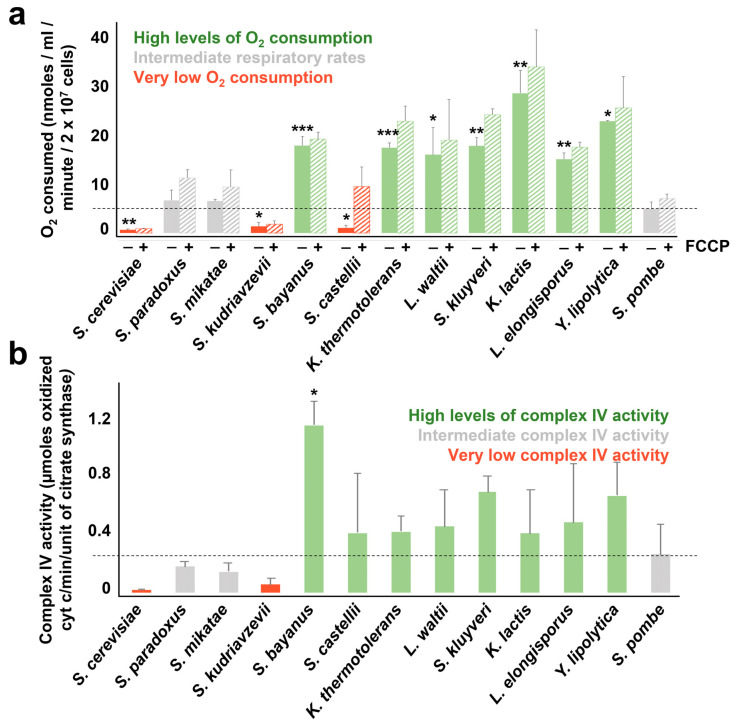
Measuring mitochondrial activity with two different approaches. (**a**) Mitochondrial oxygen consumption of whole cells showed differences in the respiratory rates of the 13 species. Oxygen consumption levels were measured in cells growing in YE5S when cultures reached an OD_600_ of 0.5. Bars represent the mean and SD from three biological replicates. Significant differences relative to *S. pombe* were determined by two-sided *t*-test (* *p* < 0.05, ** *p*  <  0.01, *** *p*  <  0.001). Maximal respiratory capacity upon addition of the uncoupler FCCP is also shown. Yeast with significantly high or low respiratory rates are highlighted in green or red, respectively. (**b**) Complex IV activity assay of isolated mitochondria. Cytochrome c oxidation was monitored at 550 nm and referenced to citrate synthase units as described in Section 2. Yeast with high, intermediate or low complex IV activity are highlighted in green, grey or red, respectively. Bars represent mean and SD from biological duplicates. Significant differences relative to *S. pombe* were determined by two-sided *t*-test (* *p*  <  0.05).

We first monitored oxygen consumption of the different yeasts grown in YE5S. As shown in Figure 3a, the levels of oxygen consumption varied a lot among the different species. While three of them displayed undetectable levels of oxygen consumption (*S. cerevisiae*, *S. kudriavzevii* and *S. castellii*), another group of yeasts had respiratory efficiencies comparable to those of *S. pombe* (*S. paradoxus* and *S. mikatae*). Seven species displayed very high levels of oxygen consumption even at these high concentrations of glucose (standard YE5S contains 3% glucose), with *K lactis* consuming 5 times more oxygen than *S. pombe* (Figure 3a). Lowering the concentration of glucose in YE5S (Figure S3a) or adding the mitochondrial uncoupler FCCP to reach maximal respiratory rates (Figure 3a) enhanced the oxygen consumption of most yeasts to different extents.

We also monitored the complex IV activity of mitochondrial-enriched fractions of the different yeasts by measuring cytochrome c oxidation. We first normalized the mitochondrial fractions by measuring citrate synthase activity (Figure S3b). As shown in Figure 3b, complex IV activity from purified mitochondria yielded similar results to those obtained with measurement of oxygen consumption of whole cells (Figure 3a). Thus, it was evident that mitochondria fractions of *S. cerevisiae* and *S. kudriavzevii* displayed very low levels of cytochrome c oxidation (in red in Figure 3b), while *S. paradoxus*, *S. mikatae,* and *S. pombe* mitochondria had intermediate levels of complex IV activity (in grey in Figure 3b). Seven yeasts’ mitochondria displayed very high levels of cytochrome c oxidation (in green in Figure 3b), as they also displayed high levels of oxygen consumption (Figure 3a). The only yeast showing different results using both methods was *S. castellii:* it displayed undetectable levels of oxygen consumption, but high levels of complex IV activity. Since the second method involves the isolation of the mitochondria-enriched fraction, we hypothesized that a repressive factor acting in whole cells is eliminated in the purified *S. castellii* mitochondria. In fact, this yeast displayed a remarkable difference in oxygen consumption in the presence or absence of FCCP (Figure 3a).

**Figure 4:**
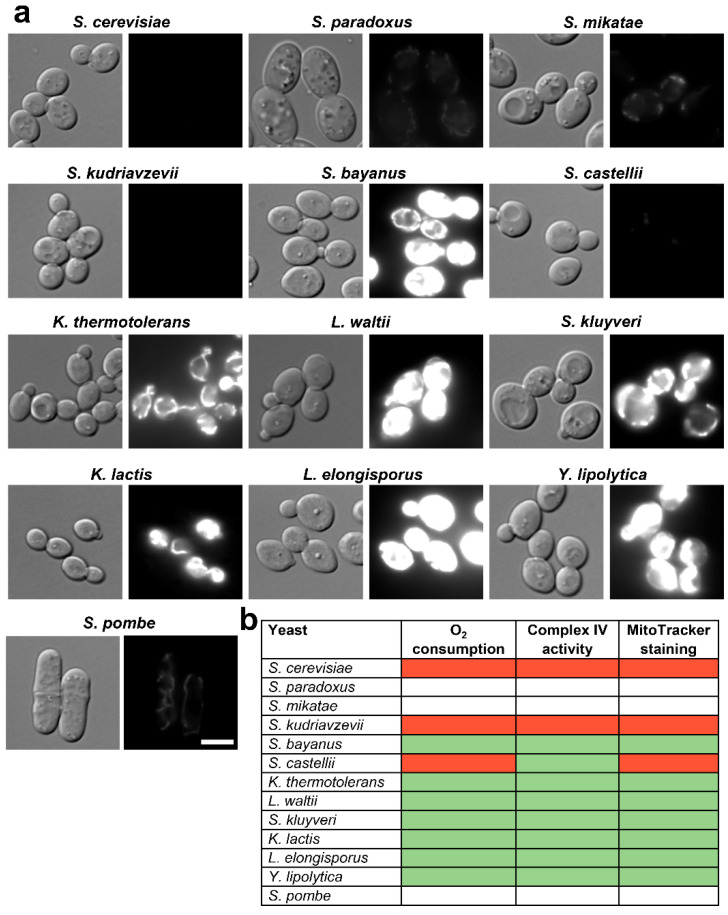
Measuring mitochondrial activity using MitoTracker Red CMXRos. (**a**) Mitochondria accumulation of the dye MitoTracker Red CMXRos was determined by fluorescence microscopy. The same cells under differential interference contrast (DIC) optics and red fluorescence are shown. Maximum and minimum levels were adjusted using Fiji. Scale bar, 5 μm. (**b**) Table comparing the results obtained by the three methodologies employed to measure mitochondrial activity: oxygen consumption, complex IV activity and MitoTracker staining. Colour code indicates respiratory rates (high: green; low: red).

Since the maintenance of the mitochondrial membrane potential (ΔΨ) is a parameter directly linked to respiratory metabolism, we used MitoTracker Red CMXRos, a ΔΨ-dependent fluorescent dye, as a third method with which to monitor mitochondrial activity. It has to be taken into consideration that dyes staining the mitochondria have to be carefully assessed depending on the cell type and may only be used to corroborate alternative methodologies to measure mitochondrial activities, since the loading and mitochondrial localization of the dye can be influenced or not by loss of ΔΨ and the dye may itself inhibit respiration [31,32,33]. MitoTracker Red CMXRos is a rosamine-based, red-fluorescent organic dye, which diffuses through the plasma membrane of live cells and accumulates within mitochondria through a charge-based affinity. A chloromethyl moiety on the structure allows the dye binding to thiol groups on proteins through this chloromethyl group, which enhances the dye’s retention. The charge-related accumulation of MitoTracker Red CMXRos is what gives the dye its usefulness as an indicator of mitochondrial membrane potential: the fluorescent intensity diminishes upon mitochondrial depolarization (for a recent review, see [34]). As shown in Figure 4 by fluorescence microscopy, the dye heavily stained the mitochondria of 7 strains, and barely incorporated into the mitochondria of *S. cerevisiae*, *S. kudriavzevii* and *S. castellii* (Figure 4 and Figure S4). These results are in complete agreement with the levels of oxygen consumption as shown in Figure 3a, and similar to those of the complex IV activity (Figure 3b). We conclude that these three methodologies are equally capable of classifying the strains regarding mitochondrial efficiency, as shown in Figure 4b.

### 3.4. Measuring Tolerance to Oxidative Stress of the Different Yeasts

We then monitored the oxidative stress tolerance of the 13 yeast species by recording the growth curves in YE5S, containing or not two concentrations of H_2_O_2_: 2 mM and 10 mM. The first concentration is capable of activating stress responses in fission yeast and triggering a survival response, which allows growth resumption after a growth arrest of 5–20 h [26]; cultures at the logarithmic phase of fission yeast are not capable of resuming growth after 10 mM H_2_O_2_ stress.

**Figure 5:**
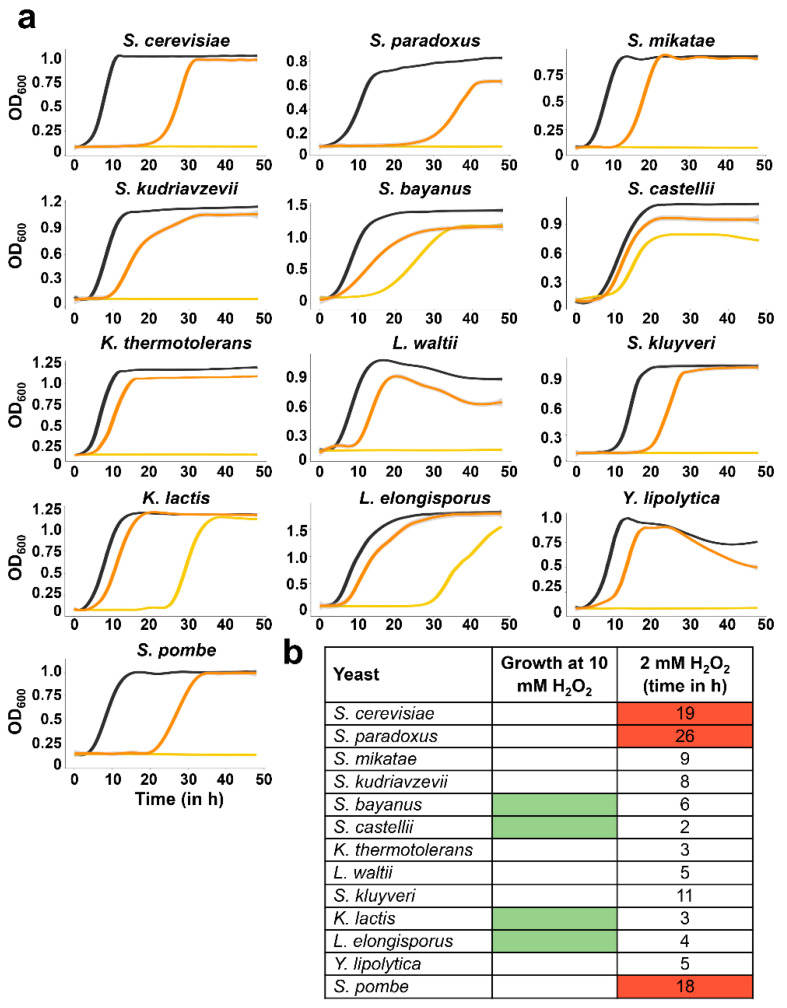
Analysis of tolerance to H_2_O_2_ in liquid cultures. (**a**) Yeasts were grown in YE5S in the absence (dark grey) or presence of 2 mM (orange) or 10 mM (yellow) H_2_O_2_. Growth rates were monitored by measuring the OD_600_ during 48 h. Each line represents the average of three independent replicates and grey shadows indicate the SD. (**b**) Table summarizing tolerance to peroxides. Second column indicates the resistant strains capable of resuming growth in the presence of 10 mM H_2_O_2_ treatment (marked in green). Third column indicates the time (in hours) each yeast needs to recover after 2 mM H_2_O_2_ treatment; in red: the three strains more sensitive to H_2_O_2_ stress.

As shown in Figure 5a, four yeast cultures were able to resume growth in the presence of 10 mM: *S. bayanus*, *S. castellii, K. lactis* and *L. elongisporus.* Similarly, these four yeasts displayed the shorter arrest time after 2 mM stress, relative to untreated conditions (3–6 h in Figure 5b). The three strains with the highest sensitivity to 2 mM stress were *S. cerevisiae*, *S. paradoxus*, and *S. pombe* (in red in Figure 5b).

## 4. Discussion

To understand the molecular events and selection processes regulating longevity, it is imperative to examine the differences in the lifespans of closely related species, within a controlled environment. Here, we present a large-scale comparative functional analysis of the relationship between mitochondrial respiration, stress tolerance, and longevity, by measuring them in 13 *Ascomycota* yeast species, spanning over 250 million years of evolution. Twelve of them belong to the hemiasocomycetes subdivision, also known as the budding yeasts, while *S. pombe* belongs to the subdivision of the archiascomycetes, which divide by fission instead of budding. In particular, the 12 budding yeasts analyzed here display similar morphology and common lifestyles, which contradicts their actual molecular divergence at the evolutionary level. Especially regarding the genomes of the 12 *Hemiascomycota* philus (all except *S. pombe*), they have lost most introns and have a reduced influence of transposable elements, and this has been hypothesized to give them a limited potential to form novel genes and gain new biological functions compared to multicellular eukaryotes [13]. As highlighted before for other biological processes [28,35,36], we show here that the evolutionary divergence of these species is correlated with the huge diversity of respiratory behaviors, tolerance to oxidative stress, and survival during the stationary phase.

*S. pombe* and *S. cerevisiae* are very distant and their genetic architectures very different; nevertheless, the fission yeast is closer, regarding the phenotypes described here, to members of the hemiasocomycetes subdivision than *S. cerevisiae* is. It is also worth pointing out that different genetic backgrounds within the same genus and species can display differences regarding respiration. That is the case for the main two *S. cerevisiae* backgrounds used by the scientific community, namely BY4741 (the one used in this study for CLS, mitochondrial efficiency and H_2_O_2_ tolerance) and W303 derivatives. BY4741 directly derives from the original S288C, while W303 has been modified by diverse crosses to several other strains [37]. Several reports have demonstrated significant differences between these two backgrounds, in particular regarding replicative lifespan [38]. In fact, we have also determined that respiration (as determined by oxygen consumption, complex IV activity and MitoTracker staining) is markedly different between both *S. cerevisiae* backgrounds, with BY4741 displaying no respiration at high glucose concentrations (3% of standard YE5S) and W303 respiring to levels comparable to *S. pombe* (Figure S5a–c). These experiments suggest that BY4741, but not W303, is subject to strong catabolite repression, since lowering concentration of glucose enhances mitochondrial respiration in this background (Figure S5d). The tolerance to H_2_O_2_ of these two backgrounds also differs (Figure S5e), while both strains seem to be long-lived (Figure S5f).

Based on the phenotypes to CLS and oxidative stress, and in their mitochondrial activity in high glucose-containing media, we have classified the 13 yeast species in 4 groups (Table 1). Group 1 includes 3 species (*K. lactis*, *S. bayanus* and *L. elongisporus*) with high mitochondrial activity and remarkable resistance to oxidative stress, as well as a long-lived phenotype. High respiratory activity, however, is not a direct indicator of longevity: as shown for Group 2 members (*S. kluyveri*, *K. thermotolerans*, *L. waltii* and *Y. lipolytica*), these species display a rather short viability at the stationary phase. We have included in Group 3 *S. mikatae*, *S. paradoxus* and *S. pombe*, which display intermediate levels of mitochondrial activity, longevity, and H_2_O_2_ tolerance. Group 4 includes three species with undetectable levels of respiration in YE5S; to our surprise, they either displayed an elongated lifespan (*S. kudiravzevii* and *S. cerevisiae*) or high tolerance to H_2_O_2_ (*S. castellii*).

As shown in Table 1, a trend found in our study is that some yeast species displaying high viability at the stationary phase are also resistant to H_2_O_2_ at their logarithmic phase (Group 1). In yeasts of this group, we hypothesize that high mitochondrial metabolism, through the production of ROS, may contribute to the activation of global anti-stress gene expression programs, causing these beneficial effects on aging and H_2_O_2_ survival, and probably enhancing other more global anti-stress capabilities. High or low respiratory rates, however, do not directly correlate with longer lifespan except for Group 1 yeasts. The Crabtree effect, described as the repression of respiration by the fermentation pathway in the presence of high glucose concentrations occurring in many yeasts [39,40], cannot explain the differences observed in this study in terms of respiration: most species of our collection are Crabtree positive yeasts, with the likely exceptions of *K. lactis* [41] and *Y. lipolytica.* This does not seem to have a direct impact on mitochondrial efficiency: while *K. lactis* displayed the highest levels of oxygen consumption, *Y. lipolytica* and five other species also had very high levels of respiration in equivalent conditions of oxygen supply and glucose concentration (Figure 3). The loss of genes encoding complex I of the electron transport chain in the mitochondrial DNA is a landmark of the *Saccharomycetaceae* genomes that differentiates them from other subgroups such as the ones of *K. lactis* or *Y. lipolytica*. Remarkably, the same loss occurred in the group of *S. pombe* [12,42]; again, this feature does not correlate with high or low mitochondrial efficiency. It is important to mention that all three methodologies used in this manuscript to measure mitochondrial activity (oxygen consumption, MitoTracker staining and complex IV activity of mitochondrial-enriched fractions) yielded equivalent results.

## 5. Conclusions

Multiple studies propose a link between increased respiration and extension of lifespan, although those studies often rely on the application of calorie restrictions and report a beneficial effect on longevity in a given model system. This effect is often considered a consequence of the activation of stress responses by signals emanating from active mitochondria, i.e., reactive oxygen species [5,11,43]. In our study, we show that the picture is not so simple when using 13 different yeast species and the same culture media. By measuring both longevity and tolerance to H_2_O_2_, we demonstrated a good correlation between them in most yeasts: long-lived species are also resistant to H_2_O_2_ stress. However, mitochondrial activity per se cannot anticipate viability at the stationary phase, with unforeseen results regarding the lifespan of heavily respiring yeast. Future studies will need to be performed to explain the alternative molecular events that account for the different lifespans of these 13 yeast species.

## Figures and Tables

**Table 1:** Four groups of yeasts based on mitochondrial activity, longevity, and oxidative stress tolerance.

Yeast Strains	Phenotypes		Mitochondrial Activity ^c^		
	Longevity by FACS ^a^	H_2_O_2_ Tolerance ^b^	O_2_ Consumption	MitoTracker Staining	CIV Activity
**Group 1**					
*K. lactis*					
*S. bayanus*					
*L. elongisporus*					
**Group 2**					
*S. kluyveri*					
*K. thermotolerans*					
*L. waltii*					
*Y. lipolytica*					
**Group 3**					
*S. mikatae*					
*S. paradoxus*					
*S. pombe*					
**Group 4**					
*S. kudriavzevii*					
*S. castellii*					
*S. cerevisiae*					

^a^ Green cells indicate high survival rates, red cells indicate low survival rates at stationary phase. ^b^ Green cells indicate high survival rates, red cells indicate low survival rates upon H_2_O_2_ stress. ^c^ Green cells indicate high mitochondrial activity, red cells indicate low mitochondrial activity, based on O_2_ consumption, MitoTracker staining or complex IV activity, as indicated.

## Data Availability

The data presented in this study are available in the article and Supplementary Materials.

## Supplementary Materials

The following supporting information can be downloaded at: https://www.mdpi.com/article/10.3390/antiox12101810/s1. Figure S1: Growth curves of 13 yeast species in different rich media; Figure S2: Determination of the CLS of the 13 yeast species by flow cytometry; Figure S3: Oxygen consumption levels at different glucose concentrations and citrate synthase activity of protein extracts; Figure S4: Comparing MitoTracker Red CMXRox staining in the 6 yeast strains with the lowest mitochondrial activity; Figure S5: Comparing phenotypes of two *S. cerevisiae* strains: W303 and BY4741.
