# Supplementary material for: Comparing Mitochondrial Activity, Oxidative Stress Tolerance, and Longevity of Thirteen Ascomycota Yeast Species
Source: Antioxidants (Basel). 2023 Sep 28;12(10):1810. doi: 10.3390/antiox12101810 (PMC10604656; doi:10.3390/antiox12101810)
Supplement: Supplementary file 1 [file antioxidants-12-01810-s001.zip › antioxidants-2566097-supplementary.pdf]

SUPPLEMENTARY DATA

Comparing mitochondrial activity, oxidative stress tolerance, and longevity of thirteen *Ascomycota* yeast species

Gröger et al.

It includes:

5 Supplementary Figures

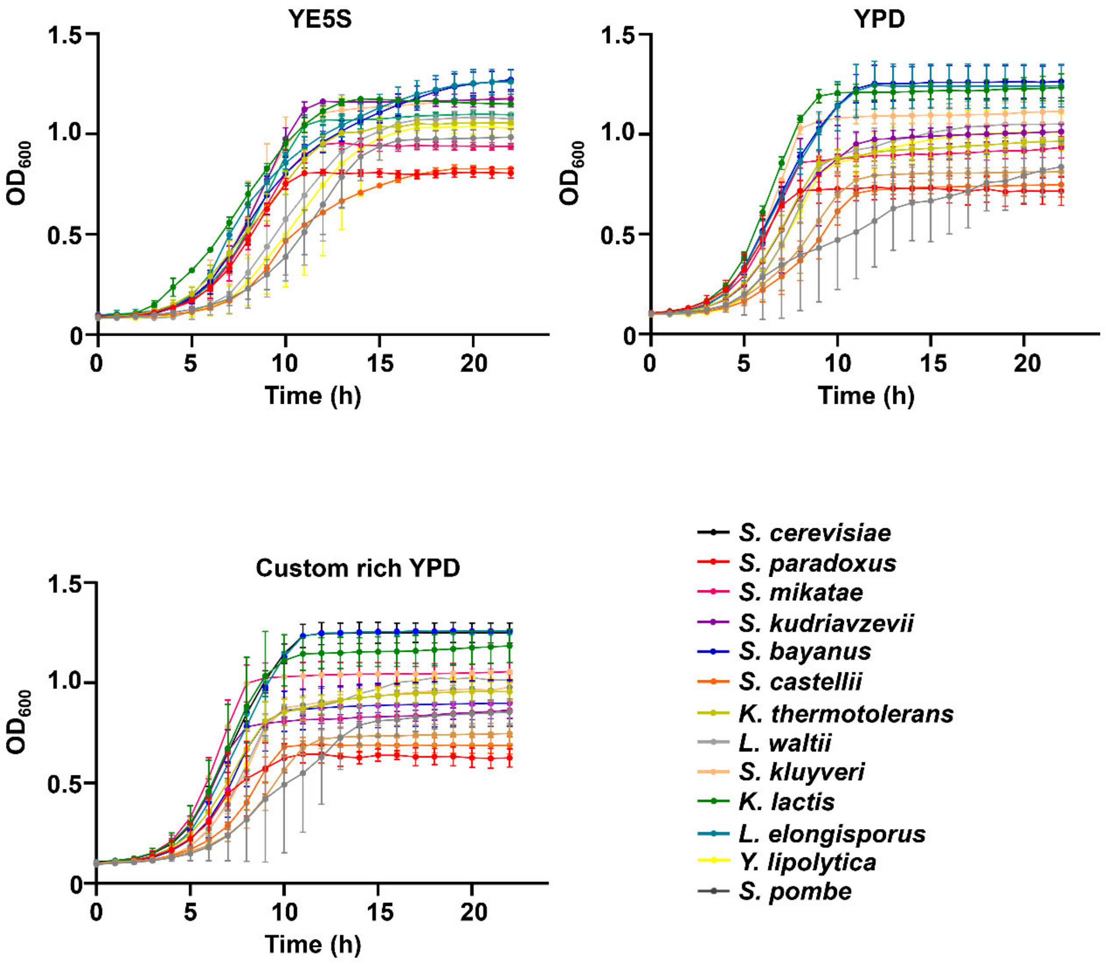

Figure S1. Growth curves of 13 yeast species in different rich media. All yeasts display comparable growth rates in YE5S, YPD, and custom rich YPD media. Cell growth was monitored measuring OD_{600} during 24 hours. Each line represents the average of three biological triplicates with error bars (SD) in the indicated yeast strains.

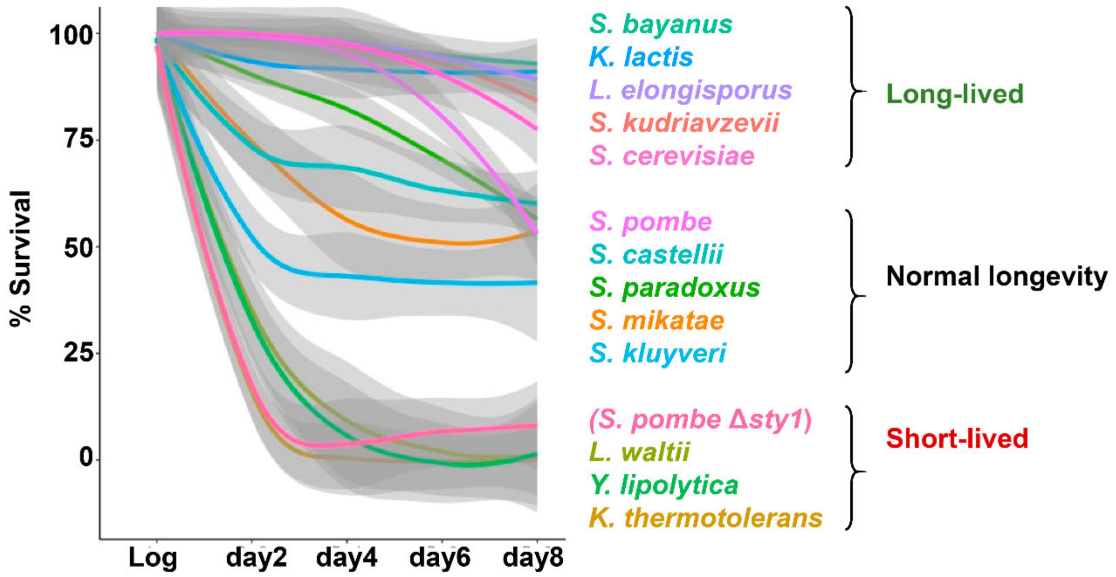

Figure S2. Determination of the CLS of the 13 yeast species by flow cytometry. Longevity of each yeast strain in YE5S media was measured for 8 days. Each line represents the average percentage of survival at logarithmic growth (Log), day 2, day 4, day 6 and day 8 of stationary phase. Lines are the average of three biological replicates and grey shadow the standard deviation (SD).

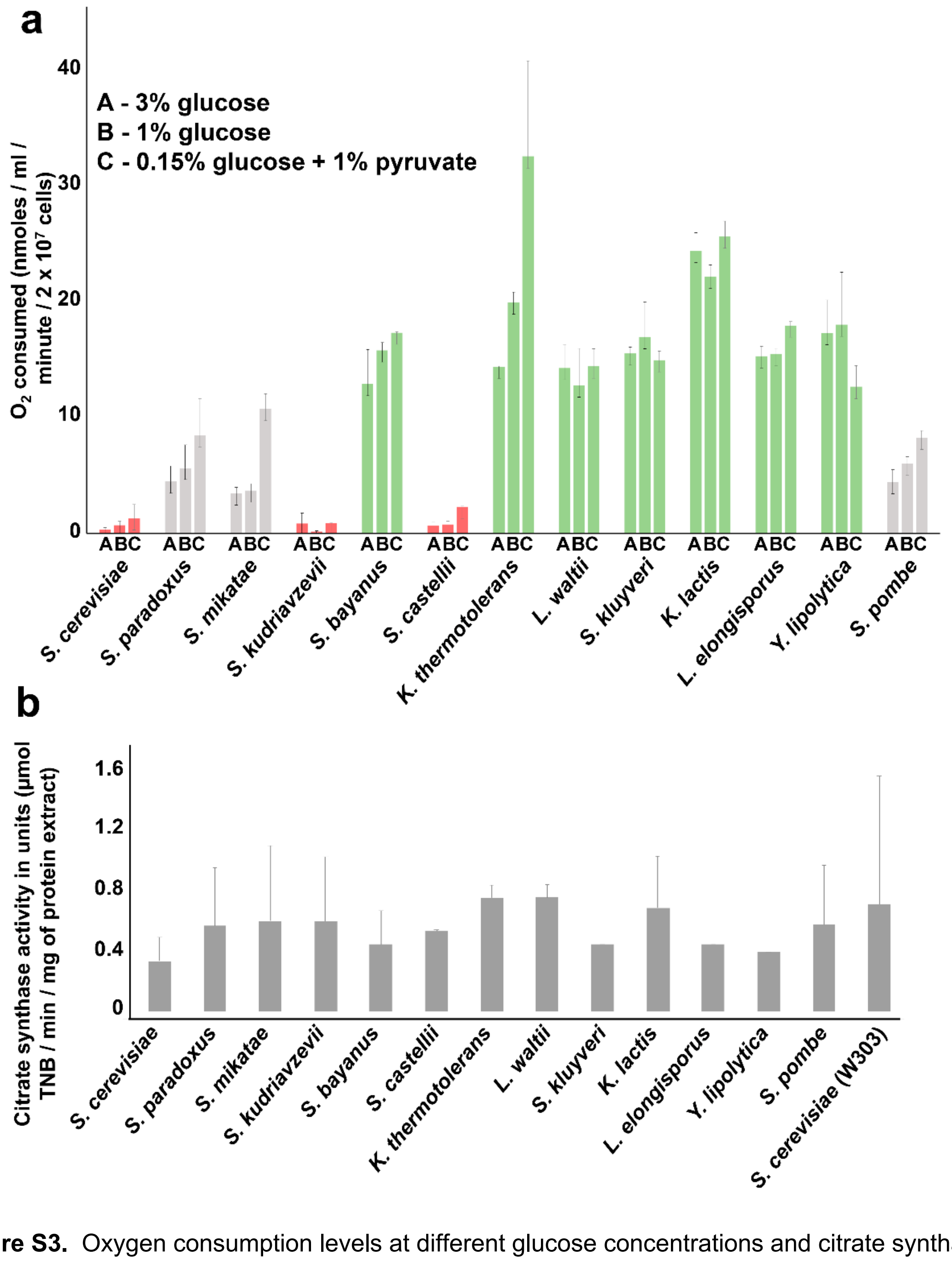

Figure S3. Oxygen consumption levels at different glucose concentrations and citrate synthase activity of protein extracts. **(a)** Mitochondrial oxygen consumption was measured in cells growing in three different media: YE5S 3% glucose, YE5S 1% glucose and YE5S 0.15% glucose + 1% pyruvate. Data from three biological replicates are shown with error bars representing standard deviation (SD). Yeast with high or low respiratory rates are highlighted in green or red, respectively. **(b)** Quantification of mitochondria-enriched fractions by citrate synthase activity. See Materials and Methods for details.

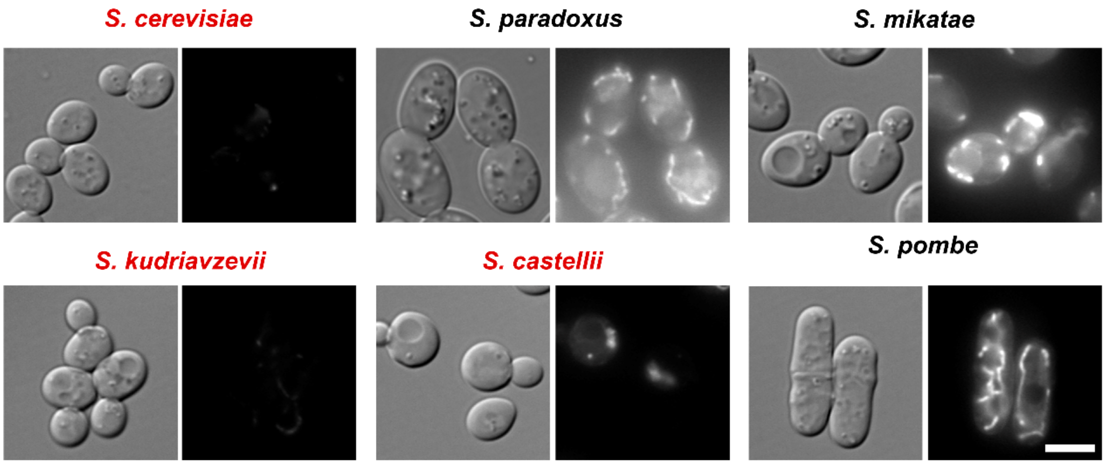

Figure S4. Comparing MitoTracker Red CMXRox staining in the 6 yeast strains with the lowest mitochondrial activity. Mitochondria of *S. cerevisiae*, *S. parodoxus*, *S. mikatae*, *S. kudriavzevii*, *S. castellii*, and *S. pombe* were stained with MitoTracker Red CMXRos and mitochondrial activity was monitored using fluorescence microscopy. Micrographs of DIC and red fluorescence are shown. Minimum and maximum levels were adjusted using Fiji. Scale bar, 5 µm. Red color highlights the strains with undetectable oxygen consumption levels.

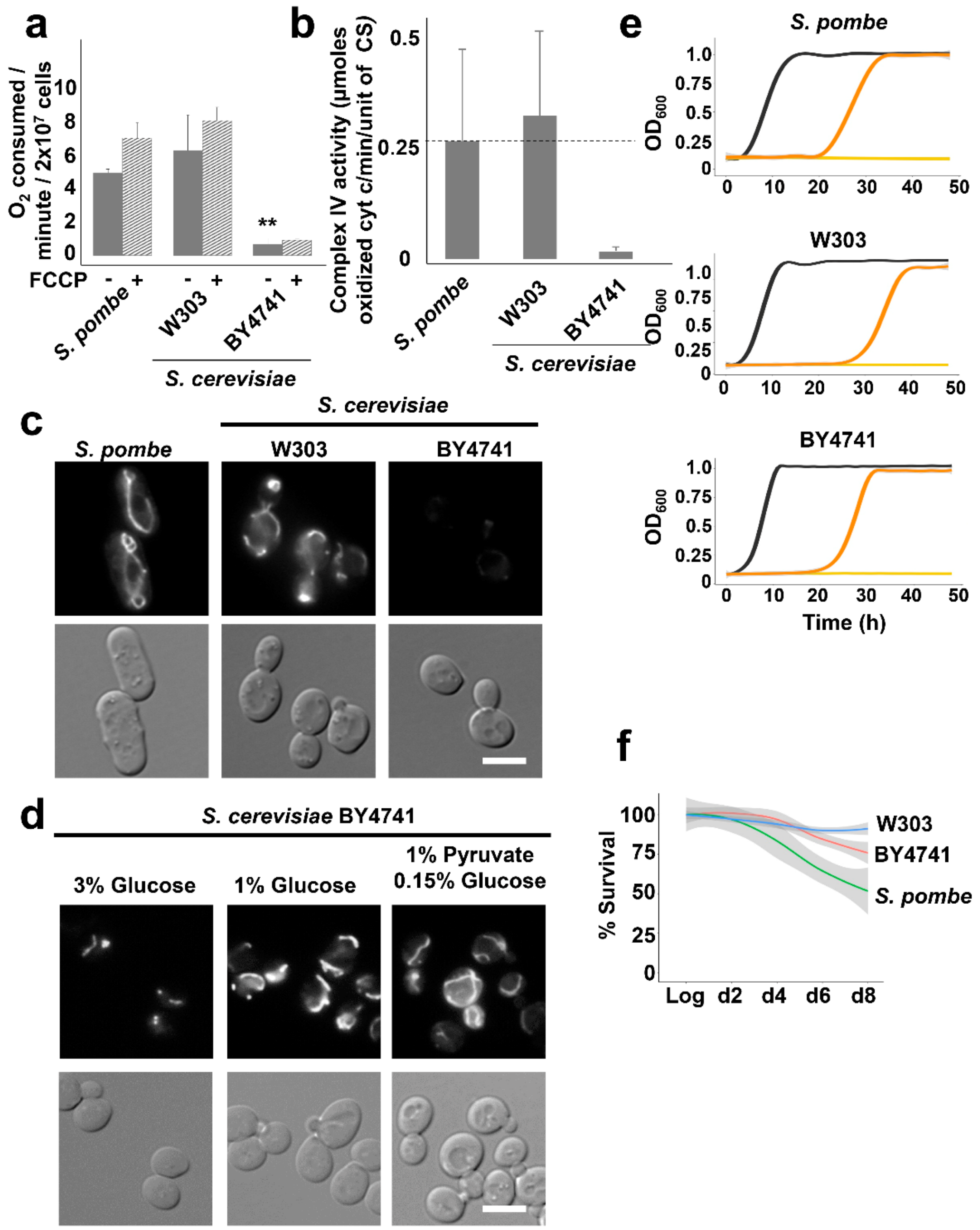

Figure S5. Comparing phenotypes of two *S. cerevisiae* strains: W303 and BY4741. **(a)** Mitochondrial oxygen consumption levels of the two *S. cerevisiae* strains and *S. pombe*. Oxygen consumption levels were measured in cells growing in YE5S 3% glucose when cultures reached an OD_{600} of 0.5. Data from three biological replicates with error bars showing SD. Significant differences between each yeast specie and *S. pombe* were determined by two-sided t-test (**$p < 0.01$). Maximal respiratory capacity upon addition of the uncoupler FCCP is also shown. **(b)** Activity of complex IV was assayed in mitochondrial enriched samples in the indicated strains. Cytochrome c oxidation was monitored spectophotometrically at 550 nm, and reference to citrate synthase (CS) units. Bars represent average of two independent

experiments with error bars showing SD. **(c)** Mitochondrial activity using MitoTracker Red CMXRos. Mitochondria of cells *S. cerevisiae* strains and *S. pombe* growing in YE5S 3% glucose were stained with MitoTracker Red CMXRos and mitochondrial accumulation was determined by fluorescence microscopy. Micrographs show DIC and red fluorescence. Minimum and maximum levels were adjusted with Fiji. Scale bar, 5 µm. **(d)** Mitochondrial activity measured by MitoTracker Red CMXRos of the *S. cerevisiae* strain BY4147 grown under different glucose concentrations. Cells of BY4741 grown in YE5S 3% glucose, YE5S 1% glucose and YE5S 0.15% glucose 1% pyruvate were stained with MitoTracker Red CMXRos. Micrographs show DIC and red fluorescence to measure mitochondrial activity. **(e)** Tolerance of *S. cerevisiae* strains to H_2O_2 in liquid cultures. Yeast cells of the *S. cereviviae* strains W303 and BY4147, and the *S. pombe* strain were grown in the absence (dark grey) or presence of 2 mM (orange) or 10 mM (yellow) H_2O_2. Growth rates were monitored by measuring OD_{600} during 48 hours. Each line represents the average of three biological replicates and grey shadow the standard deviation. **(f)** Longevity of *S. cerevisiae* strains. Longevity of *S. cerevisiae* strains BY4741 and W303, and *S. pombe* grown in YE5S 3% glucose was measured for 8 days. Each line represents the average percentage of survival at logarithmic growth (Log), day 2 (d2), day 4 (d4), day 6 (d6) and day 8 (d8) of stationary phase. Lines are the average of three biological replicates and grey shadow the SD.
